# Informing care through lived experiences: perspectives of consumers and carers regarding dietetic care for eating disorders in Australia

**DOI:** 10.1007/s40519-022-01481-9

**Published:** 2022-10-21

**Authors:** Alana Heafala, Lana J. Mitchell, Lauren Ball

**Affiliations:** 1 School of Health Sciences and Social Work, Griffith University, 1 Parklands Dr, Southport, QLD 4215 Australia; 2 Menzies Health Institute Queensland, Griffith University, 1 Parklands Dr, QLD 4215 Southport, Australia

**Keywords:** Carers, Consumers, Dietetics, Eating disorders, Person-centered care, Qualitative

## Abstract

**Purpose:**

Dietitians are important members of eating disorder treatment teams. Previous research indicates little is known about the experience of receiving nutrition care for eating disorders. This study aimed to explore the perspectives of consumers and carers regarding the care received from primary care dietitians for eating disorders.

**Methods:**

This study qualitatively explored the perceptions of individuals aged ≥ 15 years, who (i) identified as having an eating disorder or (ii) had cared for someone with an eating disorder, and had received care from a dietitian in a primary care setting. Thematic analysis was used to identify themes from interview transcripts. Synthesized member checking was utilized to assess whether the identified themes resonated with participants’ experiences. Twenty-four individuals (21 consumers, 3 carers) participated in a semi-structured interview. Seventeen participants completed member checking and all supported the identified themes and subthemes.

**Results:**

Three themes emerged inductively from the data: (1) valuing a person-centered approach to dietetic care; (2) the therapeutic alliance is central to engaging in dietetic care; and (3) sharing the complex journey.

**Conclusions:**

This study advances the understanding of the aspects of dietetic care perceived as most helpful by consumers and carers. These insights highlight the importance of person-centeredness, empathy, trust and collaboration within eating disorder care. The findings can be used by dietitians and health professionals to inform practice. Further research is needed to understand how dietitians can be supported to provide optimal nutrition care to people and families impacted by eating disorders.

**Level of evidence:**

V. Qualitative study.

## Introduction

Eating disorders (EDs) are complex mental illnesses that involve distorted thoughts about food, weight and body shape [1]. EDs impact people of all ages, genders and cultures [2], directly affecting approximately 15% of the Australian population at some point in their lives [3]. Early identification and intervention can positively shape the trajectory of illness [4]. If left undiagnosed, the disorder can become ingrained, impacting quality of life and the effectiveness of treatment [5]. 

Equipping primary health care professionals with the knowledge and skills to recognize the signs and symptoms of EDs may enhance early identification and engagement in treatment. People may be unaware they have an ED and may seek help from health professionals for seemingly unrelated health concerns [6, 7]. People may seek treatment for physical symptoms such as weight concerns, gastrointestinal complaints, bone health and infertility, contributing to EDs going undetected and untreated [5, 8]. A systematic review of 14 studies found people with EDs are more likely to receive treatment for weight concerns rather than their eating behaviors [5]. These findings suggest a lack of awareness and understanding of EDs may be preventing people from accessing the services they need. Primary care is an important setting for early identification and intervention of EDs as it is often the first point of contact people have with the health care system [9]. 

Dietitians work within multidisciplinary teams and are a key contributor to the ongoing management of EDs through the Medicare ED management plan enabling eligible Australians up to 20 subsidized dietetic consultations per year [10]. Nutrition rehabilitation is a core component of ED recovery alongside medical and psychological management [11]. Primary care dietitians assess a consumer’s weight history, nutritional beliefs, values, eating patterns and behaviors to tailor care and help foster a positive relationship with food [12]. Dietetic treatment for EDs is enhanced through continuing professional development to ensure dietitians provide safe and effective nutrition care [13]. Currently, little is known about the provision of nutrition care for EDs in primary care.

This study aimed to explore perspectives of receiving dietetic care for EDs in a primary care setting. Consumers and carers can offer valuable insight into the experience of receiving nutrition care for EDs and aspects of care they perceive as important for recovery [14]. Although perspectives of ED treatment have been examined more broadly [15], little research has explored perspectives of dietetic care. These insights can inform dietitians and other health professionals about optimizing their practice for people and families impacted by EDs [16].

## Methods

A qualitative phenomenological approach informed through the lens of pragmatism was used to explore perspectives of dietetic care. This approach allowed the researchers to seek to understand the lived experiences of consumers and carers receiving care from a primary care dietitian for EDs [17]. The recommendations outlined in the standards for reporting qualitative research checklist were used to report the findings [18].

### Sample

Purposive sampling was used to recruit eligible participants who were ≥ 15 years, (i) identified as having an ED (current or past) or (ii) had cared for someone with an ED, and had received care from a primary care dietitian. Primary care dietitians were defined as those who work within community, private practice and outpatient settings. Consumers whose experiences with dietitians were solely in an inpatient setting or specialist ED day program were not eligible. 

Potential participants were invited to participate in the study via an online link on a recruitment flyer that was circulated on social media, e-newsletters and ED organization websites in Australia. The information sheet and consent form outlined the purpose of the study and were included on the online form. Basic demographic data (age, gender), participant type, had received care from a primary care dietitian and contact details were collected to assess eligibility criteria. Participation was voluntary and online form completion implied consent. The lead researcher (AH) contacted all prospective participants via email or text message to schedule interviews at a time and mode that was preferred by the participant. 

Ethical approval was obtained from Griffith University Human Research Ethics Committee prior to data collection (ref: 2021/551). As the study included participants < 18 years and people with EDs, additional measures were taken to ensure the study was sensitive and appropriate [19]. Prospective participants aged 15-17 years were contacted (by AH) to determine their ability to make an informed decision about participating in the research. Two sets of informed consent materials were developed, one ‘addressed to’ the parent/guardian and the other to the young person [20]. A distress response protocol for interviews was developed and a trigger warning for EDs and details of support services were communicated to each participant prior to commencing the interview [19]. Consumers and carers were interviewed separately to maintain confidentiality and create a safe space for participants to disclose personal information and their experience.

### Response

Seventy-four responses (68 consenting to participate, 6 requesting further information), were received for the study within three weeks of recruitment. Twenty-four interviews were conducted between September and November 2021, until no new codes emerged and data saturation was achieved [21]. Due to a lower response from carers, all carers were invited to participate in an interview (*n* = 3). Efforts were made to recruit more carers via snowball sampling; however, no additional participants were recruited.

### Data collection

In-depth, semi-structured interviews were conducted via telephone or videoconference using a purpose-developed interview guide [22]. The guide was piloted with a researcher who was not involved in the study and two people with lived experience of EDs to assess sensitivity and appropriateness, and to identify any items that may have been overlooked by the research team [19]. Interviews were audio recorded and transcribed verbatim using Microsoft Teams and transcription software (Otter.ai). A total of 24 interviews were conducted via telephone (*n* = 15) and videoconference (*n* = 9), and lasted an average of 33 min (range 19–53 min).

To enhance trustworthiness and confirmability, an audit trail and reflective journal were used throughout the study, and to record reflections after each interview [22]. All participants received an AU$20 eGift voucher to acknowledge their time. Interview participants were selected based on participant type, age and gender identity to provide a diverse range of perspectives regarding dietetic care for EDs. The type of EDs were recorded as participants reported them in interviews rather than diagnostic criteria. 

### Data analysis

The transcribed data were uploaded to NVivo to facilitate thematic analysis. A five-phased approach was used to thematically analyze the data [23]. Synthesized member checking was used to enhance authenticity and credibility of the findings [24]. Receiving transcripts can be confronting for participants and cause embarrassment [25]. To minimize such an occurrence, the identified themes and subthemes were provided to participants via an online form. Participants were asked to assess whether the themes resonated with their experience and sufficiently represented what they had shared with the research team. This additional step provided an opportunity for participants to engage with and contribute feedback to analyzed data, months after their original interview [24]. Member checking was completed by 17 (of 24) participants in March 2022 with all supporting the identified themes and subthemes.

### Participant characteristics

Demographic details of participants are presented in Table 1. Twenty-one participants identified as having a lived experience with EDs (consumers) and three participants identified as having cared for someone with an ED (carers). Most participants were female (*n* = 23), with one male participant. Most consumers reported experiencing a restrictive-type ED including anorexia, bulimia nervosa and other specified feeding and eating disorders. Others described how their ED had changed over time, transitioning from restrictive type to binge eating type throughout the years. Two participants recalled the ED being present in childhood, with many experiencing EDs in adolescence.

**Table 1:** Characteristics of interview participants (*N* = 24) in this study of consumer and carer perspectives of dietetic care for eating disorders

Characteristic	*n*	(%)
Participant type		
Consumer	21	(87.5)
Carer	3	(12.5)
Age		
15–17 years	1	(4.2)
18–24 years	10	(41.6)
25–34 years	7	(29.2)
35–44 years	2	(8.3)
45–54 years	4	(16.7)
Gender		
Female	23	(95.8)
Male	1	(4.2)
Reported type of ED^a^		
Anorexia Nervosa	12	(57.1)
Anorexia & Bulimia Nervosa	3	(14.3)
OSFED	2	(9.5)
Transitioned/changed over time	2	(9.5)
Predominantly restrictive type	2	(9.5)
Age of onset^a^		
5–10 years	2	(9.5)
11–15 years	13	(61.9)
16–20 years	5	(23.8)
≥ 21 years	1	(4.8)
Duration of illness^a^		
0–5 years	6	(28.6)
6–10 years	8	(38.1)
11–15 years	3	(14.3)
16–20 years	2	(9.5)
> 20 years	2	(9.5)
Geographical location		
Queensland	15	(62.5)
Victoria	5	(20.8)
New South Wales	2	(8.3)
South Australia	1	(4.2)
Western Australia	1	(4.2)

*ED* Eating disorders, *OSFED* Other specified feeding and eating disorders

^a^Consumers only

## Results

### Identified themes

Three themes emerged inductively from data: (1) valuing a person-centered approach to dietetic care; (2) the therapeutic alliance is central to engaging in dietetic care; and (3) sharing the complex journey. Themes are presented with subthemes in Table 2.

**Table 2:** Themes and subthemes emerging from inductive thematic analysis

Theme	Subtheme
Valuing a person-centered approach to dietetic care	Connecting with the individual and adapting care to the needs of consumers
	Empowering consumers and carers with knowledge and skills to navigate recovery
	Taking a holistic and inclusive approach to dietetic care
	Perceiving communication and coordination between treating team, consumers and carers as beneficial for recovery
	Dietitians’ understanding, experience and expertise in EDs
The therapeutic alliance is central to engaging in dietetic care	Consumers perceiving mutual trust, empathy and rapport as integral to therapeutic alliance
	Accountability, support and validation perceived as important for ongoing dietetic care
Sharing the complex journey	Carers recognizing the signs and symptoms and advocating for help
	Consumers and dietitians needing patience and perseverance, and trusting the process of recovery
	Choosing recovery and having a support system
	Dietitians considering impact on families and reinforcing role of nutrition in recovery
	Prioritizing and balancing treatment with daily life

*ED* Eating disorders

#### Valuing a person-centered approach to dietetic care

Consumers appreciated when dietitians took their needs and preferences into consideration and engaged them in the decision-making process. Participants who felt uninvolved in their care described having difficulty trusting the dietitian and implementing nutrition strategies. Factors participants felt were important for dietitians to consider included: age; stage of recovery; cultural background and preferences; living situation; social influences such as social media; personal goals and motivations.

I think the big thing for my experience was that it wasn’t until a dietitian actually asked me, ‘What's important to you?’ or ‘What do you want to work towards?’ And my goal was, at that point, to stay out of hospital and she acknowledged that and was like, ‘Okay, well let's work with that’. (P08, consumer)

Dietitians were described as ‘invaluable’ for understanding the role of nutrition in recovery. Many participants reported being equipped with knowledge and skills they continued to use after treatment had ceased. Nutrition education regarding the importance of different food groups, metabolism and understanding how food aids recovery were perceived as important. Participants reported food monitoring, challenging self-limiting beliefs and behaviors, and collaboratively developing goals were helpful.

He [dietitian] was more balanced and helped me learn the difference between I guess (sic) healthy attitudes to wanting to eat well and exercise, versus the eating disorder thoughts wanting to be excessive with exercise and food restriction. (P19, consumer)

A holistic approach to dietetic care was valued by participants. Enquiring about other aspects of their lives such as work, university, body image concerns, family life and periods of additional stress or anxiety helped participants feel heard. Several participants described seeking a dietitian who used a weight-neutral, non-diet and health at every size (HAES) approach to care. It was recognized that this allowed them to focus on eating for health, including more foods in their diet and relying less on food rules to guide eating practices.

I think there are many dietitians out there who will happily fuel eating disorder behavior. I was just really, really lucky that I happened to end up with someone who was a HAES professional and intuitive eating approach because had she not been, I feel like my restrictive behaviors would have exacerbated tenfold… (P20, consumer)

Communication and collaboration between consumers, carers and the treating team were perceived as beneficial by all participants. Levels of communication and coordination of care varied between participants. Those who reported a high level of coordination within their team felt this positively influenced their experience of care. Some felt uncomfortable reporting information between treating team members, preferring clinicians to communicate with each other.

… It was difficult for me because I was having to relay my blood results and my weight changes to both the psychologist and dietitian, which I mean it's frustrating as a patient in any sense, but also for an eating disorder patient I don't think it's the best idea for them to give me that capacity to lie, essentially, if I wanted to. (P11, consumer)

Experience and expertise in EDs were perceived as key attributes for dietitians to possess. One participant felt a non-specialist dietitian can be just as helpful; however, most consumers felt their dietitian’s knowledge and expertise in EDs was important for recovery.

I would see someone that has good experience, I wouldn't see someone that doesn't specialize because they wouldn't understand the psychological component... (P09, consumer)

#### The therapeutic alliance is central to engaging in dietetic care

Therapeutic alliance refers to a collaborative partnership between a consumer and dietitian as they work together towards a common goal. Consumers reported empathy, rapport and understanding helped them establish trust with the dietitian and was perceived as essential for developing a therapeutic alliance.

She had such a firm, clear, understanding of the nuances of eating disorders and the complexities of eating disorders. I think for me that helped me build that sense of trust with her because she could really understand. (P01, consumer)

The therapeutic alliance was regarded as essential in creating a safe, non-judgmental space where consumers felt they could be vulnerable, openly share their struggles, victories, and other aspects of life with the dietitian. Several participants described how an alliance based on mutual trust and compassion helped to reduce feelings of shame and to develop a deeper understanding of their ED behaviors.

She [dietitian] never made me feel like I was weird, or she never made me feel bad for anything that I was doing or thinking. She was compassionate, had an understanding of, and helped me understand why I was doing the things that I was doing. (P17, consumer)

Accountability and support from the dietitian were seen as important for progressing in recovery. Although some participants reported knowing what they needed to do, they found the support provided by a dietitian helped to facilitate behavior change: “…I know everything she's telling me, but without her, I haven't been able to make that change” (P11, consumer). One participant shared how their dietitian was open and responsive to feedback regarding their communication and elements of care they did not find helpful.

I appreciate that someone who's worked in the field for quite a while… is still really open to learning and I guess learning not just from formal education and from the professionals, but also from lived experience. (P22, consumer)

Encouragement, validation, acknowledging progress and empowering consumers in their care were perceived as beneficial for the therapeutic alliance.

She was really empathetic and validating of my existing experiences and was very clear about I'm the expert of my own body and experience and that she was kind of co-working with me, rather [than] directing my input so it was very collaborative. (P20, consumer)

Three participants reported feeling unsupported by a dietitian when seeking help for EDs. This led them to view dietitians as unhelpful and they chose to navigate their own recovery and relationship with food. Others described similar experiences, however, sought help from a dietitian they could ‘see themselves working with’. Participants suggested it is important to ask dietitians questions to determine whether their approach and level of experience was appropriate for their needs and aligned with their values.

I think just owning it as well so even if you go to a dietitian and you don't gel, that's okay and you have the power to change that …you're not stuck, this is your treatment and your recovery so you can make it what you want. (P10, consumer)

#### Sharing the complex journey

Most participants described being unaware of the severity of their disorder and felt they were not ‘sick enough’ to require dietetic input. Rather their carer had initiated dietetic involvement, or they received a referral from another health professional. Two participants felt receiving dietetic care earlier may have prevented them from continuing to struggle with disordered eating.

I would have seen [the] dietitian a lot earlier I think. Because it just got worse and worse and even now, I don't have a full understanding of what it is to eat normally... (P09, consumer)

Carers described feeling ‘desperate’ and needing to advocate for help as there were often wait lists when seeking ED services. All carers described the dietitian reinforcing the importance of nutrition in recovery with one carer seeing the dietitian as an ally: “I guess it was just having somebody else, who wasn't me, to reiterate the message of what was important. I saw the dietitian as a bit of an ally for me, to help me help her” (P18, carer).

Readiness to change, having a support network and actively choosing recovery were perceived as important for engaging in treatment. Progress was often slow, with many participants preferring to go at their own pace which often required patience and perseverance from consumers, carers, and dietitians.

Looking back, I hated the dietitian appointments, at first, I hated them… I think I had to start to trust food... I kept saying to everyone, ‘I just want to take baby steps’… (P16, consumer)

ED recovery was described as ‘a full-time job’, often requiring weekly appointments with several health professionals. Some participants reported ceasing employment to focus on recovery, others took time off school or managed appointments around work and university. Participants reported prioritizing different treatments due to time constraints, competing schedules or for financial reasons.

There was a point where I just had to cut, like the dietitian got cut. Because my [EDP], which is the eating disorder plan from the government was like, you've used your 20 sessions, so I had to pay full price, and I couldn't afford that... (P08, consumer)

## Discussion

This study has advanced understanding of aspects of dietetic care that are highly valued by consumers and carers. The findings highlight that supportive, empathetic and collaborative relationships and receiving timely nutrition care from dietitians with appropriate knowledge and skills were important to consumers. This information can be used by dietitians and other health professionals to inform practice and guide workforce development.

Person-centered care was considered fundamental to participants, influencing their decision to engage in dietetic care. Listening, enquiring about consumers’ values and goals for recovery were examples of how dietitians demonstrated person-centeredness. Involving consumers in decision-making allows them to feel heard and provides an opportunity for dietitians to learn from their lived experience, which is also supported by the literature [26]. Guidelines and training and practice standards for dietetic treatment of EDs support a tailored and individualized approach to care [12, 13]. More clarity is needed regarding how dietitians can deliver nutrition interventions that support ED recovery and align with the values and desired outcomes of consumers.

Dietitians who had expertise and experience with EDs were of high importance to participants. Despite one participant having a positive experience with a non-specialist dietitian, most reported they would only see a specialist dietitian for EDs. Previous studies reveal a lack of confidence among dietitians and reluctance to provide care for EDs [27–29]. Reasons for this include a perceived lack of training provided during university, limited understanding of scope of practice and role of dietitians within the treating team, and a poor understanding of EDs and co-occurring mental health conditions [30, 31]. Ongoing professional development, education and supervision are recommended for dietitians working in this practice area [13]. A credentialing system was introduced to Australia in 2021 to recognize dietitians and mental health practitioners with the appropriate skills and knowledge to provide safe and effective care for EDs [32]. While it is too early to detect how the system has influenced practice, dietitians are likely to encounter EDs across the spectrum of care and not all will specialize in EDs or pursue the credential. It is imperative all dietitians receive adequate training in the screening, identification, assessment, and management of EDs. Investigating the ED content provided in university curricula for dietetics would establish a baseline of the ED training all dietitians receive. This information can be used to identify knowledge gaps to inform future training and workforce development.

The therapeutic alliance was perceived as foundational and facilitated ongoing dietetic care. Trust was central to the alliance and when this was lacking, participants disengaged in dietetic care. Empathy, rapport and a non-judgmental approach to dietetic care can help foster a sense of trust. Working collaboratively and aligning treatment with outcomes that are important to consumers can also encourage a positive therapeutic alliance [33]. Developing a therapeutic alliance based on mutual trust facilitates a collaborative approach to dietetic care that can be beneficial for both consumers and dietitians [34].

Treatment for EDs can be costly for consumers, carers and the health care system [35]. Carers and referrals from other health professionals were the main reason participants sought dietetic input. Interestingly, a Delphi study found only 50% of ED specialists felt people diagnosed with an ED should be referred to a dietitian [12]. This suggests consumers may not receive timely nutrition care and that dietitians should not rely on referrals alone [11, 12]. There is a need for dietitians to demonstrate how they can support people in their recovery and to communicate the value of early dietetic care in the treatment of EDs. Research investigating the effectiveness of dietetic care and documenting nutritional outcomes in practice can help demonstrate the value of dietetic involvement in ED treatment.

### Strengths and limitations

The semi-structured interviews provided rich data regarding consumer and carer perspectives of dietetic care for EDs. Qualitative research does have some limitations; however, several measures were taken to enhance the rigor and trustworthiness of the findings. Member checking demonstrated that the identified themes reflected participants’ experiences. It is acknowledged that people with atypical presentations and binge type EDs experiences of dietetic care may differ significantly from those reported in this study. There were a limited number of carer participants, targeting carer organizations directly may enhance recruitment of carers for future research.

### What is already known on this subject?

Little is currently known about the experience of receiving nutrition care for EDs. Investigating the perspectives of consumers and carers has provided rich insight into dietetic care for EDs and the elements that are most important to those receiving this care. This information can inform practice and be used to optimize health services for people and families impacted by EDs.

### What this study adds

This study has advanced understanding of aspects of dietetic care that are highly valued by consumers and carers. The findings highlight that supportive, empathetic and collaborative relationships and receiving timely nutrition care from dietitians with appropriate knowledge and skills were important to consumers.

## Data Availability

The data that support the findings of this study are available upon reasonable request from the corresponding author. The data are not publicly available due to privacy or ethical restrictions.
